# Phos-Tag-Based Analysis of Myosin Regulatory Light Chain Phosphorylation in Human Uterine Myocytes

**DOI:** 10.1371/journal.pone.0020903

**Published:** 2011-06-09

**Authors:** Hector N. Aguilar, Curtis N. Tracey, Siu Cheung F. Tsang, Justin M. McGinnis, Bryan F. Mitchell

**Affiliations:** 1 Department of Physiology Faculty of Medicine and Dentistry, University of Alberta, Edmonton, Alberta, Canada; 2 Department of Obstetrics and Gynecology, Faculty of Medicine and Dentistry, University of Alberta, Edmonton, Alberta, Canada; University of Cincinnati, United States of America

## Abstract

**Background:**

The ‘phosphate-binding tag’ (phos-tag) reagent enables separation of phospho-proteins during SDS-PAGE by impeding migration proportional to their phosphorylation stoichiometry. Western blotting can then be used to detect and quantify the bands corresponding to the phospho-states of a target protein. We present a method for quantification of data regarding phospho-states derived from phos-tag SDS-PAGE. The method incorporates corrections for lane-to-lane loading variability and for the effects of drug vehicles thus enabling the comparison of multiple treatments by using the untreated cellular set-point as a reference. This method is exemplified by quantifying the phosphorylation of myosin regulatory light chain (RLC) in cultured human uterine myocytes.

**Methodology/Principal Findings:**

We have evaluated and validated the concept that, when using an antibody (Ab) against the total-protein, the sum of all phosphorylation states in a single lane represents a ‘closed system’ since all possible phospho-states and phosphoisotypes are detected. Using this approach, we demonstrate that oxytocin (OT) and calpeptin (Calp) induce RLC kinase (MLCK)- and rho-kinase (ROK)-dependent enhancements in phosphorylation of RLC at T18 and S19. Treatment of myocytes with a phorbol ester (PMA) induced phosphorylation of S1-RLC, which caused a mobility shift in the phos-tag matrices distinct from phosphorylation at S19.

**Conclusion/Significance:**

We have presented a method for analysis of phospho-state data that facilitates quantitative comparison to a reference control without the use of a traditional ‘loading’ or ‘reference’ standard. This analysis is useful for assessing effects of putative agonists and antagonists where all phospho-states are represented in control and experimental samples. We also demonstrated that phosphorylation of RLC at S1 is inducible in intact uterine myocytes, though the signal in the resting samples was not sufficiently abundant to allow quantification by the approach used here.

## Introduction

The cellular responses mediated by protein phosphorylation are vast in number and function [1], and therefore a variety of biochemical techniques has been developed to study this important cell signaling modality [2]. Among these, western immunoblotting (WB) is the most widely utilized for the routine measurement of phospho-proteins in experimental samples after 1-dimensional sodium dodecyl sulfate – polyacrylamide gel electrophoresis (SDS-PAGE). The power and utility of this technique has recently been strengthened by the development of a dinuclear metal complex ‘phosphate-binding tag’ (phos-tag) that can be incorporated into the polyacrylamide gel matrix prior to SDS-PAGE [3], [4]. This modification of traditional SDS-PAGE, promotes a physical separation of phospho-proteins proportional to the phosphorylation stoichiometry. Thus a single protein might separate into multiple bands, each corresponding to a different phospho-state (forms of a protein containing the same number of phospho-modifications).

In traditional phospho-protein analyses by WB, the signal derived from a phospho-specific antibody (Ab) toward the target protein is normalized to a reference protein, or to the ‘total’ (‘bulk’) target protein using an Ab that does not discriminate between phosphorylated and non-phosphorylated forms. This type of analysis is normally limited to measuring changes at a single phospho-site per assay. In contrast, Mn^2+^-phos-tag SDS-PAGE permits the study of the effects of experimental treatments on the target protein across its various phospho-states by probing replica membrane with an anti-total-target protein Ab and enables the study of protein phosphorylation in the absence of phospho-protein-specific Abs. Where phospho-protein-specific Abs are available, the technique yields information regarding the distribution of specific phosphoisotypes (i.e. identical phosphorylation sites) across various phospho-states (i.e. equivalent phosphorylation stoichiometries). As such, this technique permits identification of the phospho-states corresponding to specific phosphoisotypes. A previous report using Mn^2+^-phos-tag SDS-PAGE to assess phosphorylation of myosin regulatory light chain (RLC) demonstrated the enhanced sensitivity of the technique [5]. Others have focused principally on discovery of novel phospho-states, or quantifications of single phospho-states or phosphoisotypes [6]–[8]. We have used this methodology to validate the phospho-specificity of Abs directed toward RLC in lysates derived from primary cultures of human uterine myocytes [9]. Here we quantified the changes in the phospho-states of RLC, by employing a method that does not rely on a ‘loading control’ protein, and produces vehicle-corrected data that are expressed relative to the untreated distribution to enable a direct comparison between drugs with different vehicles.

As an exemplary model, we have measured the phospho-state distribution of RLC in human uterine myocyte lysates under various experimental conditions. The contractility of uterine and other smooth muscle (SM) beds is dependent on the state of phosphorylation of RLC. In particular, phosphorylation of RLC at S19 triggers cell shortening and force production at the tissue level [10]–[14]. The primary enzyme implicated in phosphorylation of RLC at S19 is myosin RLC kinase (MLCK) [15]. The resultant phospho-S19-RLC (p19RLC), through an unknown mechanism, is associated with activation of the myosin heavy chain ATPase, which provides energy for the power stroke. Subsequent phosphorylation of T18, resulting in T18/S19-diphospho-RLC (p18p19RLC) causes enhanced activation of the myosin ATPase [16], [17]. Production of p18p19RLC might therefore be of significant importance for SM force production. This potentially important distinction between mono- (S19) and di-phosphorylated (T18/S19) proteins exemplifies the utility of a relatively simple approach to assess phospho-state changes following experimental perturbations.

A potential complication in this phospho-RLC analysis is that a single phospho-state could contain more than one phosphoisotype. Similarly, one phospho-site might appear in more than one phospho-state [18]–[20]. Therefore, the goals of this study were to 1) develop a method for quantification of phospho-state distribution data, 2) evaluate and validate this method under various treatment conditions, and 3) evaluate whether the reported changes in phosphorylation of RLC result from activity at T18 and S19 alone, or whether they might be explained by activity at other phosphorylation sites. The scheme for data analyses provided here represents a novel method for evaluating phosphorylation reactions that might yield significant insight into the integration of cellular signals at the level of the target protein.

## Results and Discussion

### Detection of Myosin Regulatory Light Chain Phospho-states in Uterine Myocyte Lysates

**Figure 1:**
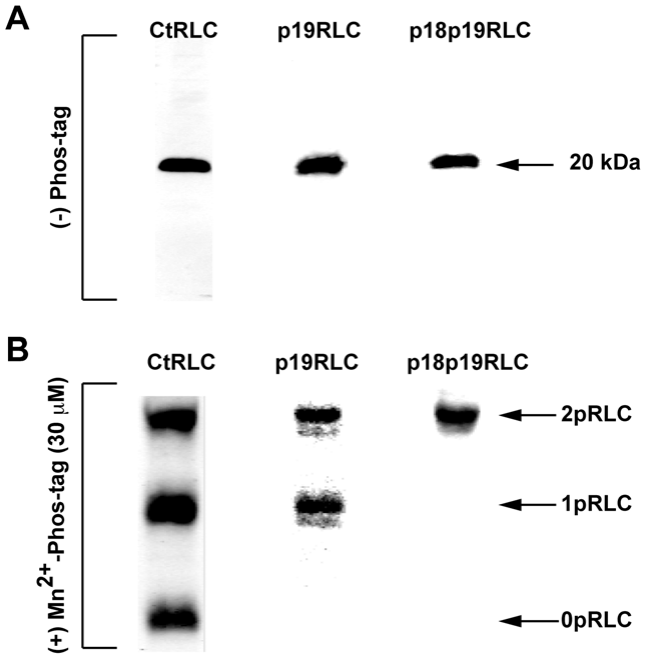
Demonstration of RLC phospho-states in human uterine myocyte lysates. **A.** WBs produced by separation of proteins in lysates from unstimulated uterine myocytes by traditional SDS-PAGE. Single lanes were loaded with ∼25 µg of protein/lane. Abs directed against the C- terminus of total-RLC (CtRLC), phospho-S19-RLC (p19RLC), and diphospho-T18/S19-RLC (p18p19RLC) identify a single prominent band of 20 kDa. **B.** WBs produced after Mn^2+^-phos-tag SDS-PAGE. CtRLC, p19RLC, and p18p19RLC Abs identified three, two, and one specific band(s), respectively. The lower, middle, and upper bands in these blots correspond to non-, mono-, and di-phosphorylated RLC, denoted as 0pRLC, 1pRLC, and 2pRLC.

Several studies have shown that three distinct phospho-states of RLC are detectable in SM preparations [5], [18]–[25]. These three phosphoisotypes most likely correspond to unphosphorylated, mono-phosphorylated (S19), and diphosphorylated (T18/S19) RLC. To ensure preservation of phospho-modifications during cell lysis, we harvested total cellular proteins by trichloroacetate precipitation (see methods). After traditional SDS-PAGE separation, Abs directed toward the C-terminus of total-RLC (CtRLC, no phospho-specificity), phospho-S19-RLC (p19RLC), and phospho-T18/S19-RLC (p18p19RLC) recognize only a single band of 20 kDa on WB replica membranes (Figure 1A). In contrast, when uterine myocyte protein lysates are separated by Mn^2+^-phos-tag SDS-PAGE these three Abs yield distinct banding patterns. The CtRLC Ab (Figure 1B) produces three distinct bands in lysates from unstimulated uterine myocytes. These data do not provide information regarding the phosphoisotypes contained within each band. Thus, we have chosen ‘0pRLC’, ‘1pRLC’, and ‘2pRLC’ as the terminology to emphasize phosphorylation stoichiometry irrespective of the phosphoisotypes represented.

The p19RLC Ab detects two phospho-RLC phospho-states and does not detect the 0pRLC state. For clarity, we have used ‘0pRLC^T^’, ‘1pRLC^T^’, and ‘2pRLC^T^’ to denote measurements obtained by the CtRLC Ab, and the notation ‘1pRLC^19^’ and ‘2pRLC^19^’ for those reported by the p19RLC Ab. To verify that the upper of these three bands corresponds to 2pRLC, we probed a replica membrane with an anti-p18p19RLC Ab.

### Rationale and Experimental Approach for Parallel Quantification of RLC Phospho-states

In order to quantify these three phospho-states across various treatment groups, we have considered that the bands in each lane detected by the CtRLC Ab represent a ‘closed system’, such that an enhancement of one of the three phospho-states must be reflected by an equivalent diminution of one (or both) of the others, and vice versa. Use of this ‘closed system’ concept has several advantages that will be discussed in a later section that will address its validation.

To demonstrate quantifiable changes in the phospho-state distribution of RLC, we treated primary cultures of uterine myocytes with pharmacological agents likely to alter to alter phosphorylation of RLC. RhoA and rho-kinase (ROK) are known to regulate uterine contractility [26]–[30]. Specifically, rhoA-ROK activation reduces RLC phosphatase (MLCP) activity, and therefore increases intracellular phospho-RLC concentrations. To manipulate this pathway pharmacologically, cells were treated with glycyl-H-1152 (g-H, rho-kinase inhibitor) and calpeptin (Calp, rhoA activator). We also treated uterine myocytes with oxytocin (OT), which is the most potent physiological stimulant for this cell type.

**Figure 2:**
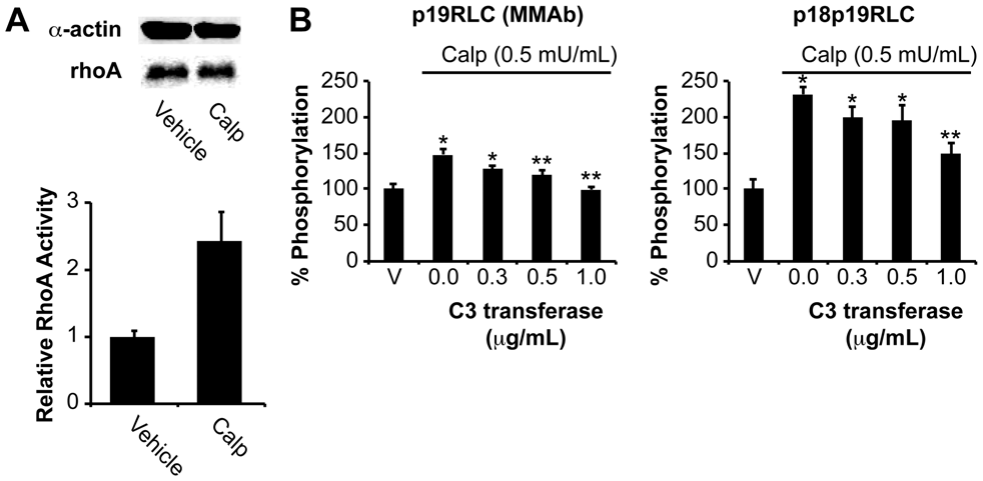
Validation of increased rhoA activity and phosphorylation of RLC by calpeptin. **A.** Estimation of GTP-bound ‘active’ rhoA in uterine myocytes treated with Calp or vehicle (DMF) for 15 min (n = 2). RhoA content was quantified relative to α-actin loading control, and used to correct rhoA activity data. Error bars represent standard deviations. **B.** Quantification of pRLC and ppRLC in uterine myocytes treated with Calp and with increasing concentrations of a cell-permeable rhoA inhibitor (C3 transferase) by ICW (n = 4). * and ** indicate significant differences from vehicle or Calp alone (second histogram), respectively. Calp (calpeptin); rhoA activator (0.5 mU/mL). p<0.05 in all cases as determined by one-way ANOVA followed by Tukey test.

Calp has been shown to specifically activate rhoA in fibroblasts cultures, possibly by influencing a protein tyrosine-phosphatase upstream of rhoA [31], [32]. To confirm that Calp increases rhoA activation in uterine myocytes, we quantified the GTP-bound ‘active’ rhoA from myocytes treated with Calp. Figure 2A confirms that Calp (0.5 mU/mL, 15 min) induces rhoA activation (2.43±0.43 fold of vehicle, n = 2) in cultured uterine myocytes. Further, we evaluated whether rhoA activation by Calp was associated with increased phosphorylation of RLC by the in-cell western technique (see methods). Figure 2B shows that Calp strongly induces diphosphorylation of RLC in comparison to monophosphorylation. The Calp-induced enhancement in diphosphorylated RLC can be attenuated by using a rhoA inhibitor (C3 transferase), again confirming that Calp activates rhoA. The cell-permeable ROK inhibitor g-H (1 µM, 30 min) was selected due to its high potency and selectivity for its target relative to other commercially available pharmacologic inhibitors [33]–[35]. We have previously demonstrated the optimal time course and concentrations for OT in this experimental system [9].

### Quantification of Phospho-state Distributions

**Figure 3:**
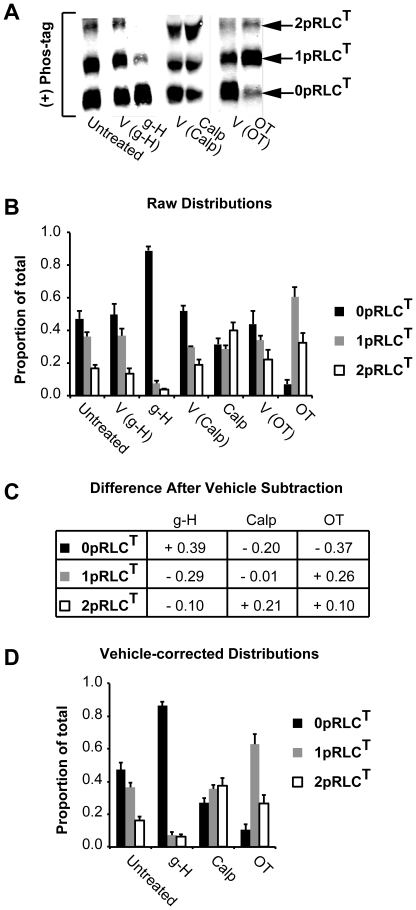
Quantification of RLC phospho-state distribution. Panels A-D correspond to WB data derived using an Ab directed toward the C-terminus of RLC. **A.** Representative WBs demonstrating RLC phospho-states separated by Mn^2+^-phos-tag SDS-PAGE. Uterine myocytes were lysed and total protein was harvested after the following treatments: untreated, or treated with g-H, Calp, OT, or with their corresponding vehicles. **B.** Quantification of bands identified in panel A (untreated; n = 12, g-H; n = 5, Calp; n = 8, OT; n = 7). The signals derived from 0pRLC^T^, 1pRLC^T^, and 2pRLC^T^ are expressed as the proportion of their sum total within each lane. **C.** Absolute magnitude of the difference in each phospho-state caused by the treatments in comparison to the corresponding vehicle (‘+’ indicates enhancement relative to vehicle, ‘−’ indicates diminution relative to vehicle). **D.** Vehicle-corrected distribution data obtained by combining the data in panel C to the untreated group distribution. g-H (glycyl-H-1152); ROK inhibitor (1 µM). Calp (calpeptin); rhoA activator (0.5 mU/mL). OT; oxytocin (100 nM). All data are shown as means ± SEMs. The corresponding numerical data are compiled in Table 1.

This analysis has two primary goals: 1) isolating the effect of the treatment of interest from any vehicle-induced change in the phospho-state distribution, and 2) facilitating comparisons across treatments by expressing them relative to a common reference point (untreated distribution). Figure 3A shows representative WBs obtained for the treatment groups described. Distribution data were obtained by expressing the signal integrated intensity at each band position as a fraction of their sum total (e.g. [signal 0pRLC^T^]/([signal 0pRLC^T^]+[signal 1pRLC^T^]+[signal 2pRLC^T^]), etc.), and averaging these proportions for n = 5–8 samples. This ‘sum method’ amounts to performing an ‘in-lane’ normalization, in place of that commonly achieved by using a loading control. We propose that this ‘in-lane normalization’ method has several advantages that are discussed in a subsequent section. Note that the WBs have been intensified for all figures so that all three bands are visible, where possible. This is achieved by restricting the upper and lower boundaries of pixel intensity that are shown by the quantification software. The quantitative estimates of band intensity are completely independent of these manipulations. The blots presented here did not exhibit pixel saturation, and therefore have not exceeded the upper limit of the scanner in detecting higher protein loads.

Figure 3B demonstrates the raw phospho-RLC proportional distributions measured by the CtRLC Ab. In the untreated distribution (n = 12), approximately 47±4% of the RLC pool is represented by 0pRLC^T^, 36±2% by 1pRLC^T^, and 16±3% by 2pRLC^T^. Next, we corrected for the effects of the vehicles by subtracting the vehicle-induced changes from those of the corresponding treatments. The net treatment-induced changes are presented in Figure 3C and these data were used to calculate the effect of each treatment relative to the untreated distribution in Figure 3D. This provides a common reference and enables comparisons across treatments (for numerical data, see Table 1). Relative to the untreated distribution, g-H (n = 5) promotes significant reduction in phospho-RLC, as evidenced an enhancement in the proportion of 0pRLC^T^ to 86±2% from 47±4% in untreated samples, whereas 1pRLC^T^ and 2pRLC^T^ fell to 7±2% and 6±2% respectively. Calp (n = 8) treatment causes a specific increase in 2pRLC^T^ from 16±3% to 38±8%, in agreement with the increase in p18p19RLC shown in Figure 2. This enhancement in 2pRLC^T^ is accompanied by a corresponding reduction in 0pRLC^T^ from 47±4% to 29±6%, but no change in 1pRLC^T^ (from 36±2% to 34±4%). These finding suggest that activation of rhoA, and presumably ROK, is associated with a specific increase in the intracellular concentrations of diphosphorylated RLC. OT appears to enhance both 1pRLC^T^ and 2pRLC^T^, perhaps favouring the former (final proportions of 54±11% and 36±10%, respectively).

**Table 1:** Changes in Phosphorylated RLC Proportions in uterine myocytes.

		% of total RLC		
	N	0pRLC	1pRLC	2pRLC
Untreated	12	47±4	36±2	16±3
g-H (1 µM)	5	86±2*	7±2**	6±2***
OT (100 nM)	7	10±2*	54±11	36±10***
OT+g-H	4	19±7*	80±8**	1±1***
OT+ML7 (25 µM)	4	20±8*	66±4**	14±6
OT+ML7+g-H	4	40±4	51±6**	9±2
Calp (0.5 mU/mL)	8	29±6*	34±4	38±8***
Calp+g-H	3	76±12*	33±15	0±0***
Calp+ML7	3	37±3	37±3	26±0
Calp+ML7+g-H	2	40±0	34±2	26±1

All data are shown as mean ± SEM.

*, **, and ***indicate significant differences (p<0.05) compared to 0pRLC, 1pRLC and 2pRLC in the untreated group, respectively.

The data shown in Figure 3C illustrate that significant quantitative differences might result from the omission of a vehicle correction step. In particular for OT, the 2pRLC^T^ state appears to be strongly induced by the vehicle, and most likely results from the mechanical stimulation of cells by pipetting the treatment solution. The short duration of treatment (20 sec) prevents this stimulatory effect from returning to basal levels. Importantly, the OT vehicle does not reduce 0pRLC^T^ as OT does (Figure 3B and C). Thus, ignoring the effect of the OT vehicle underestimates the true extent of phosphorylation of RLC induced by OT.

In summary, these data are normalized in three successive ways: 1) in-lane normalized to correct for variations in protein loading and signal intensity, 2) vehicle normalized to ascertain that the measured effect can be attributed to the drug or treatment of interest, and 3) normalized to the untreated distribution to permit comparisons across treatments.

### Validation of the ‘Closed-System’ Concept

The method for data normalization of a closed system described above has advantages over normalization to an in-lane loading control. Specifically, the use of a traditional loading control might not be feasible in Mn^2+^-phos-tag acrylamide gels for several reasons. First, the physical separation of phospho-states along the vertical dimension of the gels increases the likelihood that the loading control protein overlaps with a band corresponding to the protein of interest. Second, if it is phosphorylated, the loading control protein might dissociate into two or more bands and might complicate identification and quantification of loading control and the protein of interest on the same blot. Despite these potential problems, to validate the ‘closed system’ concept we performed a traditional loading control quantification of the phospho-states detected by the CtRLC Ab using SM α-actin as our in-lane reference.

**Figure 4 pone-0020903-g004:**
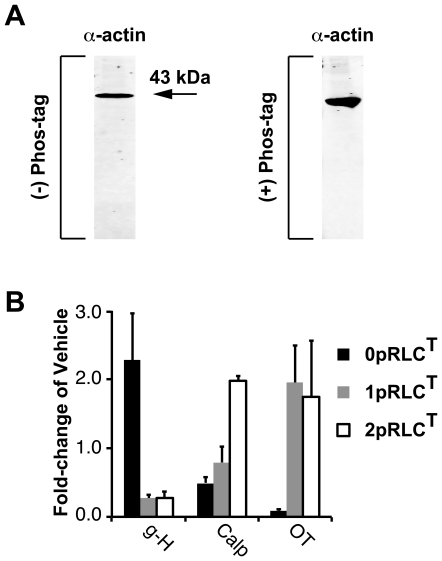
Validation of phospho-state data quantification using a loading control. **A.** WB for α-actin after traditional and Mn^2+^-phos-tag SDS-PAGE separation of proteins in lysates from uterine myocytes. In both blots, the anti-α-actin Ab recognizes only a single band. **B.** Each band in panel B was first normalized for α-actin, then corrected for the vehicle. The data are shown as fold-changes relative to vehicle for g-H (1 µM), Calp (0.5 mU/mL), OT (100 nM). All data are shown as means ± SEMs (n = 4 in all groups).

Figure 4A demonstrates that the α-actin signal remains localized to a single position after traditional and Mn^2+^-phos-tag SDS-PAGE separation without any evidence of phosphorylation under our experimental conditions. Though we were fortunate to have chosen α-actin, finding a loading control protein that does not suffer from at lease one of the above-mentioned problems might prove difficult. If a single-channel detection method is used, WB quantification might be unfeasible. Where dual-channel detection is used for segregating the loading control and target protein signals, the primary-Ab-secondary-Ab-fluorophore combinations must be of high specificity. If used appropriately, the ‘closed system’ method for data quantification proposed here circumvents all of these difficulties by eliminating the need for measuring a loading control protein.

For this validation, the signals for 0pRLC^T^, 1pRLC^T^, and 2pRLC^T^ in the vehicle and treatment lanes were normalized individually to the in-lane α-actin signal, without expressing them as the proportions of the sum-total of signals as was performed to obtain Figure 3B. The α-actin-normalized fold-changes relative to vehicle are shown in Figure 4C. Qualitatively, these data are in agreement with those in Figure 3D. ROK inhibition with g-H promotes an increase in 0pRLC^T^ relative to 1pRLC^T^ and 2pRLC^T^. RhoA activation with Calp causes a large increase in 2pRLC^T^. OT treatment causes an enhancement of both 1pRLC^T^ and 2pRLC^T^ that is reflected as a dramatic reduction in 0pRLC^T^.

To provide a direct quantitative comparison between the Figure 4C and the in-lane sum method above, we computed the fold changes in the proportion of 0pRLC^T^, 1pRLC^T^, and 2pRLC^T^ in the treatment groups relative to their proportions in the vehicle groups. No significant differences between the ‘closed system’ (‘sum method’) and the traditional loading control methods were observed. Both methods show that g-H treatment causes an increase in 0pRLC^T^ (sum method: 1.79±0.05, α-actin: 2.28±0.69 fold of vehicle), and a reduction in 1pRLC^T^ (sum method: 0.20±0.05, α-actin: 0.27±0.05) and 2pRLC^T^ (sum method: 0.26±0.07, α-actin: 0.28±0.09). Calp causes increased 2pRLC^T^ (sum method: 2.13±0.40, α-actin: 1.99±0.06). OT causes virtually complete phosphorylation of the pool of RLC, as evidenced by near abolition of 0pRLC^T^ (sum method: 0.16±0.02, α-actin: 0.09±0.03) and an increase in 1pRLC^T^ (sum method: 2.13±0.40, α-actin: 1.95±0.55) and 2pRLC^T^ (sum method: 1.47±0.18, α-actin: 1.74±0.82). The remarkable agreement between these α-actin normalized data and those in Figure 3D for these measurements demonstrate that the in-lane normalizations performed for the data in Figure 3 provide similar information to traditional loading controls.

### Analysis of p19RLC Phosphoisotype Distribution

**Figure 5:**
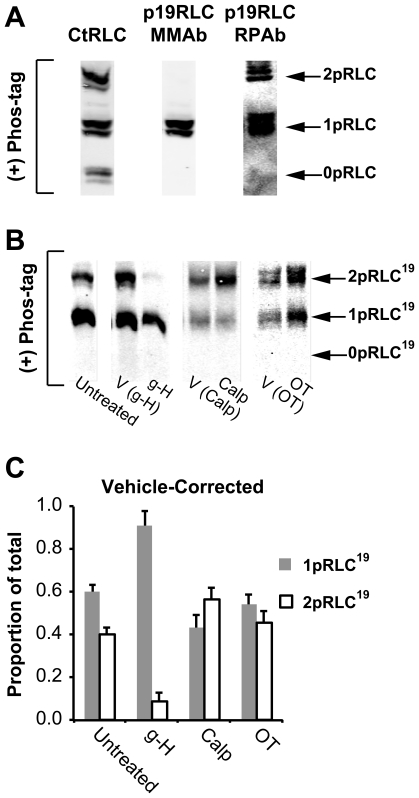
Demonstration of Phospho-S19-RLC phospho-state distribution in uterine myocytes. **A.** Detection of 0pRLC, 1pRLC and 2pRLC separated by Mn^2+^-phos-tag SDS-PAGE with an Ab toward the C-terminus of RLC (CtRLC) and two Abs (mouse MonoclonalAb [MMAb] and rabbit PolyclonalAb [RPAb]) directed toward phospho-S19-RLC. **B.** Representative WB demonstrating RLC phospho-states separated by Mn^2+^-phos-tag SDS-PAGE and probed using PRAb from panel A. Uterine myocytes were lysed and total protein was harvested after the following treatments: untreated, or treated with g-H, Calp, OT, or with their corresponding vehicles. **C.** Vehicle-corrected distribution data for 1pRLC^19^ and 2pRLC^19^ obtained by normalizing the data in panel A to the untreated group distribution. g-H (1 µM, n = 5). Calp (0.5 mU/mL, n = 10). OT (100 nM, n = 10). All data are shown as means ± SEMs. The corresponding numerical data are compiled in Table 2.

We next focused attention on the phosphoisotype composition of each of the phospho-states following Mn^2+^-phos-tag separation. In preliminary experiments, we validated the specificity of two anti-phospho-S19-RLC Abs by WB after Mn^2+^-phos-tag SDS-PAGE separation (Figure 5A). These Abs were derived from mice (monoclonal – ‘p19RLC (MMAb)’), or rabbits (polyclonal – ‘p19RLC (RPAb)’). The p19RLC (MMAb) was unable to detect the 2pRLC band present in blots probed with the CtRLC Ab and the p19RLC (RPAb). The most likely explanation for this finding is obstructed binding of the Ab in the presence of phospho-T18-RLC. Still, this Ab was useful for quantifying monophosphorylation of RLC specifically, as was demonstrated in Figure 2. Anti-total-protein Abs might also suffer from obstructed binding. Here, we selected the CtRLC Ab on the basis of published evidence suggesting phosphorylation of RLC at sites concentrated in the first 20 amino acids of the N-terminal region and the possibility of such interference was minimized [18]–[20]. We utilized the p19RLC (RPAb) to quantify phosphorylation of RLC at S19 in subsequent experiments.

One important distinction between the data provided by the p19RLC and the CtRLC Abs is that the p19RLC data does not represent a closed system, since it is unable to provide information about 0pRLC or phospho-states that do not include phospho-S19. Therefore, the use of p19RLC Ab in this setting cannot assess quantitative changes in phospho-states, but can provide information about whether the proportions of phosphoisotypes (containing phospho-S19-RLC) within each phospho-state have changed. In Figure 5C, the y-axis corresponds to the proportion of the in-lane signal that is made up by 1pRLC^19^ or 2pRLC^19^ relative to their sum (i.e. [signal 1pRLC^19^]/([signal 1pRLC^19^]+[signal 2pRLC^19^]), etc.). For numerical data, see Table 2.

**Table 2:** Changes in Relative Proportions of Phospho-S19-RLC Isotypes in uterine myocytes.

		% of phospho-S19-RLC	
	N	1pRLC	2pRLC
Untreated	12	60±3	40±3
g-H (1 µM)	5	91±7**	9±4***
OT (100 nM)	10	54±4	46±5
OT+g-H	4	62±10	39±15
OT+ML7 (25 µM)	6	62±33	39±22
OT+ML7+g-H	6	71±3**	29±4
Calp (0.5 mU/mL)	10	43±6**	57±5
Calp+g-H	2	77±1	23±1
Calp+ML7	4	55±4	45±4
Calp+ML7+g-H	3	57±5	43±5

All data are shown as mean ± SEM.

** and ***indicate significant differences (p<0.05) compared to 1pRLC and 2pRLC in the untreated group, respectively.

In the untreated group, the relative proportions of 1pRLC^19^ and 2pRLC^19^ are 60±3% and 40±3%, respectively. g-H treatment causes a shift in these proportions to 91±7% and 9±4%, suggesting that ROK inhibition causes specific loss of 2pRLC containing phospho-S19-RLC. However, the data provided earlier in Figure 3D clarify that both 1pRLC and 2pRLC are markedly reduced with g-H. In contrast, Calp changes the weighting of 1pRLC^19^ and 2pRLC^19^ to 43±6% and 57±5%, respectively, reflecting the specific enhancement in 2pRLC seen in Figures 2 and 3D. The corresponding values for OT are 54±4% and 46±5% indicating that OT induces both 1pRLC^19^ and 2pRLC^19^ to a similar extent, even if the total cellular content of 1pRLC is larger, as evidenced by the data in Figure 3D.

### MLCK and ROK Contribute to OT- and Calp-induced changes in Phosphorylation of RLC

By applying the above methods, we next evaluated the changes in phosphorylation of RLC that occur upon stimulation with OT and Calp treatments in the presence of cell-permeable inhibitors of ROK and MLCK (g-H and ML7 [34], respectively). These data are compiled in Tables 1 and 2. The data in Table 1 suggest that MLCK inhibition with ML7 results in a reduced ability of OT and Calp to cause phosphorylation of RLC, evidenced by reduced levels of 1pRLC^T^ and 2pRLC^T^ relative to OT alone, and reduced 2pRLC^T^ synthesis relative to Calp alone. Interestingly, ROK inhibition with g-H appears to completely abolish the ability of either stimulant to induce 2pRLC^T^. In the case of OT+g-H, the reduced 2pRLC^T^ is accompanied by an accumulation of 1pRLC^T^ relative to OT alone. In contrast, the effect of g-H on Calp has little to no effect on 1pRLC^T^, but results in an accumulation of 0pRLC^T^ relative to Calp alone. These data suggest that the mechanisms utilized by OT and Calp to enhance phospho-RLC concentrations are different. However, both stimulants utilize ROK to induce 2pRLC^T^. The data in Table 2 suggest that ML7 does not affect the relative proportion of phospho-S19-RLC isotypes stimulable by OT or Calp, However, in agreement with Table 1, g-H causes a loss of 2pRLC^19^ relative to 1pRLC^19^.

### Phosphorylation of Serine-1 of RLC is inducible by Phorbol Esters

In addition to S19- and T18/S19-modified phosphoisotypes for RLC, other phospho-sites located at both the N- and C-termini of RLC have been discovered *in vitro* [18]–[20]. These findings give rise to the possibility of phospho-states greater than 2pRLC [36]. However, the data presented here thus far (Figures 1 and 3) and other published evidence suggest that only three phospho-states exist. ([5], [9], [21], [22], [37]) Previous biochemical evidence from turkey gizzard SM suggested that protein kinase C (PKC) was capable of phosphorylating S1, S2, or T9 of RLC [20], [38]–[40]. Furthermore, phosphorylation of the PKC target sites prevented subsequent MLCK phosphorylation of RLC at S19, and vice versa. In addition, they demonstrated a reduction in the actin-activated myosin ATPase activity upon phosphorylation of RLC by PKC. Thus, PKC-mediated phosphorylation of RLC might impact myocyte function. To assess whether these changes occurred in human uterine myocytes, we studied the effects of phorbol-12-myristate-13-acetate (PMA), a PKC activator, on the phospho-state distribution of RLC. Surprisingly, we identified additional phospho-states for RLC that correspond to phosphorylation sites distinct from T18 or S19.

**Figure 6:**
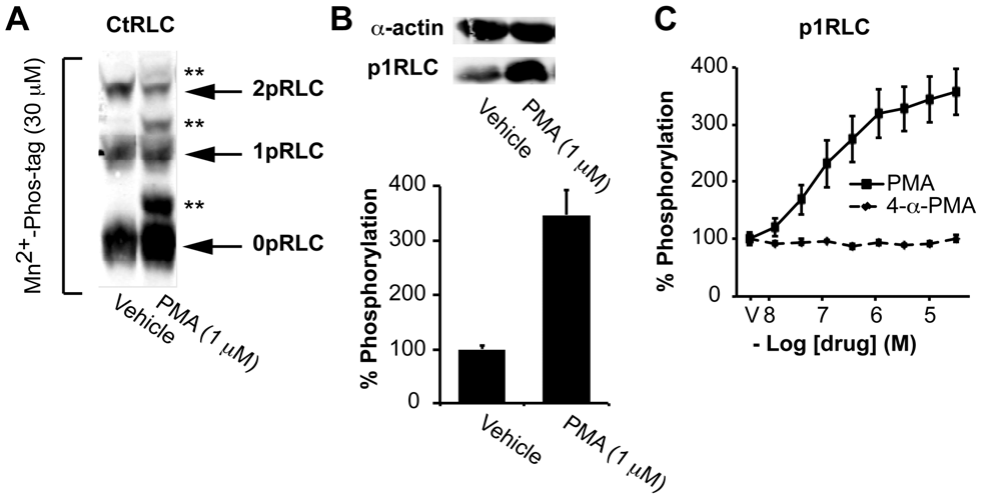
PMA induces phosphorylation of RLC at S1 and alters mobility of phospho-RLC during phos-tag SDS-PAGE. **A.** WBs produced by Mn^2+^-phos-tag separation of lysates from uterine myocytes treated with PMA (1 µM) or vehicle. The membranes were probed with an Ab directed against the C- terminus of total-RLC (CtRLC). 0pRLC, 1pRLC, and 2pRLC correspond to non-, mono-, and di-phosphorylated RLC. The bands labeled with ** exhibit unexpected mobility in the Mn^2+^-phos-tag acrylamide matrix. **B.** Traditional WBs demonstrating phosphorylation of RLC at S1 (p1RLC) in uterine myocytes treated with PMA. **C.** Quantification of p1RLC concentrations in uterine myocytes treated with PMA and 4-α-PMA (negative control) by the in-cell western method. In **B** and **C**, the data represent 4 independent experiments and are presented as means ± SEM.

Figure 6A demonstrates the separation of lysates treated with PMA (1 µM, 30 min) by Mn^2+^-phos-tag SDS-PAGE. We noted the appearance of three bands in at positions slightly above those we previously identified as 0pRLC, 1pRLC and 2pRLC, such that the CtRLC Ab cross-reacted with six distinct bands. We hypothesized that these unidentified bands might correspond to phosphoisotypes of RLC that exhibit altered mobility in the Mn^2+^-phos-tag matrix. As such, these bands might represent 1pRLC, 2pRLC, and 3pRLC corresponding to phosphoisotypes modified at a site other than T18 or S19. However, without knowledge of the specific phosphoisotypes corresponding to these intermediate bands, the quantitative information provided by the preceding analysis is difficult to interpret. Thus, instead we turned our attention to identifying a candidate phosphorylation site that might account for these additional phosphoisotypes.

We used an Ab directed toward phospho-S1-RLC (p1RLC) to determine if phosphorylation at S1 was inducible by PMA. Figure 6B shows WB data that confirms the induction of p1RLC with PMA, and is not affected by additional OT stimulation. We used the in-cell western assay to demonstrate (Figure 6C) that PMA induction of p1RLC is concentration-dependent, suggesting activation of intracellular signalling systems. Further, to distinguish between specific and nonspecific effects due to detergent-like properties of phorbol esters, we selected 4-α-PMA as negative control since this agent is ineffective at activating PKCs [41]. As shown in Figure 6C, treatment with 4-α-PMA did not result in increased concentrations of p1RLC, confirming the specificity of the PMA treatment.

**Figure 7:**
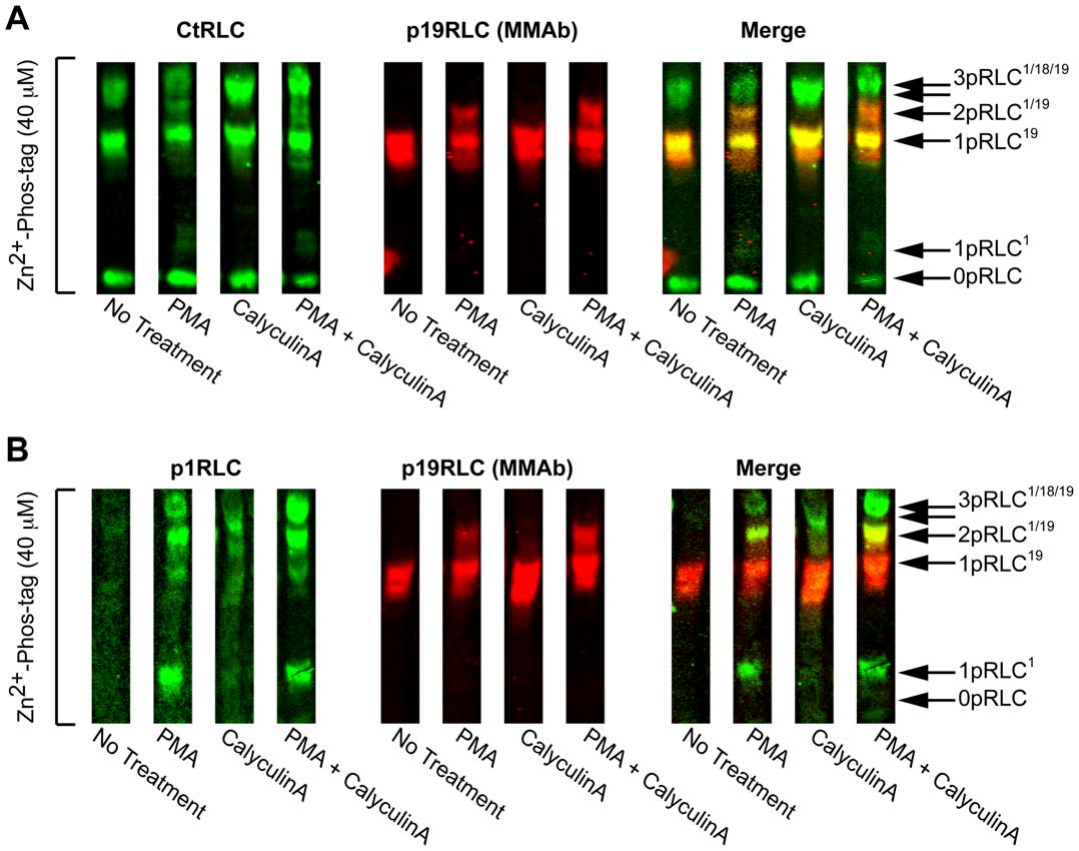
Advanced phos-tag analysis of phosphorylated RLC in PMA-treated uterine myocytes. **A** and **B.** WBs produced by Zn^2+^-phos-tag (40 µM) separation of lysates from untreated samples, or samples treated with PMA (1 µM), CalyculinA (20 nM), or with both. In both A and B, the red signal corresponds to a mouse monoclonal Ab (MMAb) targeting p19RLC that is detected independently of the green signals (CtRLC in panel A, and p1RLC in panel B). A yellow signal indicates overlap of the individual green and red signals. 0pRLC, 1pRLC, 2pRLC and 3pRLC denote non-, mono-, di-, and tri-phosphorylated RLC. The superscripts denote the position of the phospho-modification: 1: S1, 18: T18, 19: S19. The unlabeled arrow below 3pRLC^1/18/19^ corresponds to 2pRLC^18/19^ that is particularly prominent in lysates treated with CalyculinA alone.

Thus far, we have focused on the alkaline Mn^2+^-phos-tag method. Subsequent attempts to improve resolution of the unidentified bands from 0pRLC, 1pRLC and 2pRLC by Mn^2+^-phos-tag were inconsistent. Recently, an improved method of separation using Zn^2+^ instead of Mn^2+^ ions in the phos-tag matrix and an alternate buffer set for electrophoresis was developed [42]. We used this advanced Zn^2+^-phos-tag method to study the PMA-induced changes in the RLC phospho-states. In order to detect as many phospho-states as possible, we studied the effects of PMA and of the protein phosphatase 1 and 2A inhibitor, calyculin A. To elucidate the phosphoisotypes contained within each phospho-state, we used the MMAb directed toward p19RLC since it is detected independently using a secondary Ab directed towards murine primary Ab whereas the CtRLC and p1RLC (both RPAbs) are detected using a secondary Ab directed towards rabbit polyclonal Abs. Figure 7A demonstrates that the CtRLC Ab recognizes more than 3 bands in the presence of PMA, whereas phosphatase inhibition with calyculin A enhances 1pRLC and 2pRLC, but does not create any additional phospho-states. When uterine myocytes are challenged with both PMA and calyculin A, a band corresponding to a 3pRLC state can be discerned, and most likely corresponds to RLC phosphorylated at S1, T18, and S19. Figure 7B shows that the intermediate bands produced by PMA with or without calyculin A contain p1RLC, and suggests that phosphoisotypes of RLC containing phospho-S1 move more rapidly through the phos-tag matrices than those containing phospho-S19 with equivalent phosphate stoichiometries. They also suggest that there exists a 3pRLC state corresponding to phospho-S1/T18/S19-RLC that is inducible with PMA treatment. Thus, it appears that S1, T18, and T19 can exist simultaneously, rather than in mutual exclusivity to one another. However, we cannot exclude the possibility that sites other than S1, T18, and S19 have been induced by PMA, in the absence of other phospho-site-specific Abs. To clarify this possibility, future studies might benefit from the use of phos-tag SDS-PAGE in combination with other techniques such as mass spectrometry [43].

### Physiological Relevance of RLC Phospho-state Distribution Measurements

In the present work, we treated three phospho-states of RLC as a closed system based on strong evidence that other states are unlikely to exist physiologically. This rationale is in agreement with our data (Figures 1,3–5) and with studies performed in rat uteri demonstrating the three phospho-states for RLC (0pRLC, 1pRLC, and 2pRLC) as determined by two-dimensional analyses [23], [25]. Our results also agree with kinetic data regarding MLCK activity suggesting the potential for phosphorylation of nearly the whole pool of RLC during a physiological stimulus [25]. In particular, OT was shown to induce phosphorylation in approximately 80% of the pool of RLC molecules available in rat uterus [25] compared to >90% phosphorylation in our data. In contrast, it appears that a much smaller portion of the RLC pool is phosphorylated at rest in vascular SM [5]. Further, in OT-stimulated uterine myocytes from rats it appears that diphosphorylation is quantitatively more prominent than monophosphorylation [24]. Our data suggest that human uterine myocytes might respond similarly to OT with respect to mono- and diphosphorylation of RLC at S19. This validated methodology can be used to explore potentially significant differences between experimental conditions, and is likely also applicable to studying tissue differences. This type of information could be of key importance in the design of pharmacological agents that will have specificity for a single tissue or a specific physiological situation.

In our early experiments, we assumed that all phosphoisotypes with equivalent phosphate stoichiometries comigrate in the phos-tag acrylamide matrix. However, the data in Figures 6 and 7 suggest this assumption is incorrect. Under some experimental conditions, some phosphoisotypes might exist that do not conform to these migration characteristics. We demonstrated that phosphoisotypes corresponding to p1RLC exhibit altered mobility in the phos-tag matrix in comparison to p19RLC. This behaviour might facilitate further biochemical characterization of these phosphoisotypes.

Some *in vitro* data suggest that PKC-mediated phosphorylation of RLC results in reduced myosin ATPase activity and impaired S19 phosphorylation by MLCK [18]–[20], [39], [44]. However, subsequent experiments using an *in vitro* motility assay for myosins isolated from various sources demonstrated that PKC phosphorylation of RLC could not induce movement of myosin beads along actin cables on its own, and also did not alter the rate of movement of the myosin beads after MLCK-mediated phosphorylation of RLC [45]. However, the majority of available data regarding PKC actions on RLC are often irreconcilable regarding the specific site(s) of action and subsequent physiological relevance. The inconsistencies might stem from differences in model species and experimental approaches. Whatever the source of variability, the utility of PKC-mediated phosphorylation of RLC for therapeutic use in humans remains unclear. The p1RLC phosphoisotypes inducible by PMA (Figure 7) appear to make up only a small proportion of the total RLC signal present. Co-incubation of PMA with calyculin A appears to improve the incorporation of p1RLC into a greater proportion of the RLC pool, but it is unclear whether these pharmacologic manipulations are of any physiological significance. Still, it is interesting that p1RLC is inducible in intact myocytes, suggesting the presence of an intact signalling system aimed at phosphorylating a poorly characterized phospho-site on RLC. In the interest of uncovering novel therapeutic strategies for reducing smooth muscle contractility, this finding warrants further study.

## Materials and Methods

### Ethics Statement

The protocol to obtain biopsies from the lower segment of the uterus at the time of cesarean section was reviewed and approved by the Human Ethics Review Board of the University of Alberta and the Ethics Review Board of Alberta Health Services, the provider of healthcare services in the region. A research nurse at the Royal Alexandra Hospital in Edmonton obtained informed and written consent from each patient.

### Primary Myometrial Cell Cultures

This methodology has been detailed previously [9]. Briefly, Myometrial biopsies were obtained from the lower segment of the uterus from non-laboring patients at term (37–40 weeks) gestation during elective caesarean section. Biopsies were diced then incubated in HBSS (Gibco, (Invitrogen, Carlsbad, CA)) containing 1% antimycotic/antibiotic (Gibco), 2 mg/mL collagenase (Sigma-Aldrich, St. Louis, MO), and 200 ng/mL DNase I (Roche, Laval, QC) in a total volume of 10 mL for 1 hr at 37°C with shaking. After 1 h, the debris was allowed to settle, the supernatant discarded, and the remaining tissue was incubated in 10 mL of freshly prepared HBSS (+ enzymes) at 37°C while shaking overnight (O/N). The dispersed cell mixture was filtered through a 100-µm cell strainer, centrifuged at 2000×*g* for 5 min, and washed twice with PBS. Isolated uterine myocytes were grown in DMEM (Gibco) supplemented with 10% FBS (Gibco) and 1% antimycotic/antibiotic (Gibco) at 37°C in humidified 5% CO_2_/95% air. Cell cultures were grown to 80–100% confluence in a single well of a 6-well plate post-isolation, then in T25 and T75 flasks (Ultident, St. Laurent, QC). Prior to experiments, the myocytes are seeded at 600 cells/mm^2^ in 6-well plates and allowed to adhere and equilibrate O/N. The next morning, the cells were washed once with pre-warmed DMEM containing no additives, and then placed into the incubator in DMEM without additives for 2–4 hrs before treatment. All stimulants, pharmacologic agents, and drug diluents (DMSO, DMF, and H_2_O) were diluted in DMEM without additives to the final concentration prior to experiments. Oxytocin (1 mM, sterile ddH_2_O), glycyl-H-1152 (g-H, 5 mM, DMSO), ML7 (5 mM, DMSO), and phorbol-12-myristate-13-acetate (PMA, 10 mM, DMSO) were obtained from Calbiochem (EMD Chemicals, Gibbstown, NJ, USA). 4-α-PMA (10 mM, DMSO) was purchased from Santa Cruz Biotechnology Inc. (Santa Cruz, CA, USA). Calpeptin (Calp, 400 mU/µL, DMF) and the cell permeable rhoA inhibitor (C3 transferase, 1 mg/mL, H_2_O) were purchased from Cytoskeleton, Inc (Denver, CO, USA). Calyculin A (200 µM, DMSO) was purchased from Cell Signaling (Beverly, MA, USA). Uterine myocytes were treated for 20 sec with OT, 30 min with g-H, ML7, PMA, 4-α-PMA, or Calyculin A and 15 min with Calp, as determined by optimization experiments.

### Traditional and Phos-tag SDS-PAGE

Briefly, total cellular proteins were recovered by trichloro-acetate precipitation (20% TCA and 10 mM DTT in ddH_2_O), recovered into microtubes, and centrifuged at 18,000 *g* for 20 min. The protein pellets were washed several times with acetone-DTT (10 mM), allowed to air dry, then resuspended in 75 µL of 1× SDS-loading buffer by boiling for 10 min. 15 µL of each sample was loaded per lane (approximately 25 µg protein/lane) on Tris-Glycine-SDS minigels. Electrophoresis was carried out by standard methods, in 15% acrylamide gels. In addition, two methods of phos-tag (NARD Institute, Ltd., www.phos-tag.comSDS-PAGE were performed. The first method is described in [3], [9]. We used 30 µM Mn^2+^-phos-tag in 12% acrylamide gels, and performed electrophoresis in a manner identical to traditional SDS-PAGE. The second method of phos-tag SDS-PAGE is detailed in [42]. In this case, we used 40 µM Zn^2+^-phos-tag in 8% acrylamide gels and performed electrophoresis in the modified buffer set indicated in [42]. After electrophoresis, the divalent cations were removed from both types of phos-tag gels by incubation with transfer buffer containing 2 mM EDTA for 15 min. The gel contents were transferred to nitrocellulose for 1.5 hrs at 100 V by standard methods.

### Western Immunoblotting

This methodology has been detailed previously [9]. The blotting membranes were incubated for 1 hr at R/T in odyssey blocking buffer (mixed 1∶1 with PBS), then incubated overnight with primary Abs diluted in blocking buffer∶PBS supplemented with 0.1% Tween-20. The final primary Ab dilutions were: CtRLC 1∶1000 (LS-C81207, Rabbit PAb, LifeSpan BioSciences, Seattle, WA, USA), p19RLC 1∶200 (#3671, Rabbit, PAb, Cell Signaling), p19RLC 1∶2000 (#3675, Mouse, MAb, Cell Signaling), p18p19RLC 1∶500 (#3674, Rabbit, PAb, Cell Signaling), p1RLC 1∶2000 (MP3461, Rabbit, PAb, ECM Biosciences), rhoA 1∶200 (#610990, Mouse, MAb, BD Biosci., Mississauga, ON, Canada), and α-actin 1∶4000 (sc-56499, Mouse, MAb, Santa-Cruz). Secondary Abs conjugated to IRDye 800CW (anti-rabbit, Li-COR) or Alexa Fluor 680 (anti-mouse, Molecular Probes, Invitrogen) were diluted in blocking buffer: PBS supplemented with 0.1% Tween-20 and 0.01% SDS and used at a final dilution of 1∶20,000. Membranes were scanned and analyzed using an Odyssey® IR (Li-COR) scanner with imaging software version 3.0. Ab signals were analyzed as integrated intensities of regions defined around the bands of interest.

### In-Cell Westerns

This method was described in detail previously [9]. The final antibody dilutions were: p19RLC 1∶1000 (#3675, Mouse MAb, Cell Signaling), ppRLC 1∶500 (#3674, Rabbit PAb, Cell Signaling), p1RLC 1∶2000 (MP3461, Rabbit PAb, ECM Biosciences). An anti-rabbit or anti-mouse secondary Abs (1∶1000, IRDye 800CW, Li-COR) were used for detection. Ab signals were normalized to cell loading controls (DRAQ5 (1∶10,000, Biostatus Ltd.) and Sapphire700 (1∶1000, Li-COR)). Microplates were scanned and analyzed using an Odyssey® IR scanner using Odyssey® imaging software version 3.0, with an offset of 4.0 mm. Results are expressed as percent relative responses (means ± SEM) compared to vehicle-treated controls.

### Measurement of RhoA Activity by G-LISA

Relative rhoA activity was estimated by a commercially available ELISA-based assay (‘G-LISA^TM^’, BK-124, Cytoskeleton Inc.) according to the manufacturers’ specifications. Briefly, uterine myocytes were treated with 0.5 mU/mL Calp for 15 min, and then harvested by lysis in ice-cold buffer (provided). An aliquot of these samples was retained for protein content estimation by ADV02 reagent (Cytoskeleton, Inc.), and the rest was flash frozen in N_2_(l), and stored at −80°C until needed. The frozen samples were thawed and prepared for the assay as specified. Approximately 25–30 µg total protein was used per well, and each sample was assayed in duplicate. The measured optical density measured at 490 nm was expressed relative to vehicle control. These data were normalized for total-rhoA content by performing WB analysis in parallel with the rhoA activity assay. Preliminary experiments comparing uterine myocyte lysates to purified, constitutively active rhoA (G14V) positive controls demonstrated that our measurements were within the linear range of the assay.

### Statistical Analysis

All results were expressed using means ± S.E.M. In-cell western data were analyzed using one-way ANOVA, followed by a post-hoc Tukey test. Two-tailed independent t-tests were used to analyze rhoA activation and WB data, and phospho-RLC proportion data in Tables 1 and 2. A p-value<0.05 was used to establish statistical significance.
